# Optimizing the Tribological Performance of Graphite–Resin Composites: The Role of High Crystallinity, Nano Morphology, and Hydrophobic Surface Modification

**DOI:** 10.3390/nano15211655

**Published:** 2025-10-30

**Authors:** So-jung Baek, Yeo-jin Tak, Da-hyun Yu, Seong-yeon Park, Do-hyun Um, Kwang-youn Cho

**Affiliations:** 1 Korea Institute of Ceramic Engineering and Technology, Soho-ro, Jinju-si 52851, Gyeongsangnam-do, Republic of Korea; baeksj626@naver.com (S.-j.B.); jeany5004@naver.com (Y.-j.T.); rydh0925@kicet.re.kr (D.-h.Y.); ssyeon@kicet.re.kr (S.-y.P.); 2 SGO Ltd., 28-10, Nandongseoro 113beon-gil, Namgong-gu, Incheon 21695, Republic of Korea; dh92@sgoilless.co.kr

**Keywords:** graphite-resin composites, hydrophobic coupling, crystallinity, tribological

## Abstract

Graphite, with its layered structure and weak van der Waals bonding between graphene nano layers, exhibits excellent self-lubricating properties. Natural graphite, characterized by high crystallinity, and artificial graphite, with relatively low crystallinity, exhibit distinct friction behaviors and structural differences, which significantly influence the performance of graphite–resin composites as solid lubricants. This study investigates the effects of natural/artificial graphite ratios and hydrophobic silane coupling treatment on the oil impregnation behavior, friction coefficient, wear stability, and microstructural changes in graphite–resin composites. Under a vertical load of 88,260 N and surface pressure of 50 MPa, the impregnated graphite–resin composites demonstrated low friction coefficients and stable wear behavior. SEM analysis revealed well-preserved microstructures, and Raman spectroscopy confirmed the formation of stable lubrication films through the I_D_/I_G_ ratio, indicating graphene exfoliation. The results indicate that natural graphite provides dense structures and stable friction, while artificial graphite enhances oil impregnation but leads to unstable friction behavior.

## 1. Introduction

Graphite is a well-known solid lubricant with a turbostratic layered structure, in which graphene nanosheets can easily slide along the weakly bonded van der Waals planes, thereby exhibiting excellent self-lubricating behavior [1,2,3,4]. Owing to its high thermal conductivity and low thermal expansion in the in-plane direction, graphite suppresses local temperature rise and improves thermal shock resistance, ensuring durability under repeated frictional conditions [5,6]. However, under high-load and low-speed motion, the relatively low intrinsic strength of graphite necessitates the development of graphite–metal hybrid materials, in which oils or greases are typically impregnated to reduce frictional energy losses [1,7].

Recently, graphite powders have been dispersed in polymer matrices to produce graphite–resin composite lubricants with porous structures that allow effective oil impregnation and retention [8,9,10]. During sliding, graphite forms a graphene-based lubricating film, while the impregnated oil generates a secondary lubrication layer, together reducing both friction coefficient and wear. The incorporation of C_60_ fullerene has also been reported to decrease friction while enhancing elasticity, strength, and modulus via load transfer mechanisms [11,12,13]. Furthermore, graphite addition to phenolic resin-based friction materials has been shown to stabilize the friction coefficient by reducing velocity sensitivity, whereas flake graphite in epoxy resins promotes the formation of lubricating films that mitigate sudden wear [11,12]. Nevertheless, the influence of graphite crystallinity, particle morphology, and surface characteristics on tribological behavior, as well as the interfacial wettability and compatibility between oils and graphite–resin composites, remains insufficiently studied [1,8,13,14].

The present study aims to determine the optimal mixing ratio of natural and artificial graphite by evaluating the mechanical and tribological properties of composites. To enhance interfacial compatibility, graphite powders were surface-modified via silane coupling treatment, which improved oil impregnation and retention. The friction and wear characteristics were systematically investigated using a pin-on-disk tribometer, while a face-on-face configuration was employed to examine the tribological performance of graphite–metal hybrid composites under high-load continuous motion.

## 2. Experimental

### 2.1. Sample Preparation

Natural and artificial graphite powders were selected as the fillers for the resin-bonded solid lubricants, and a novolac-type phenolic resin was chosen as the binder. RUBIA 6800 15W-40 oil(Navimro, Seoul, Republic of Korea) was selected as the impregnating lubricant. To improve the wettability and compatibility between the lubricant and the resin-bonded solid lubricant, hydrophobic silane coupling was applied [13,15,16]. Table 1 shows the general properties of the raw materials used.

**Figure 1:**
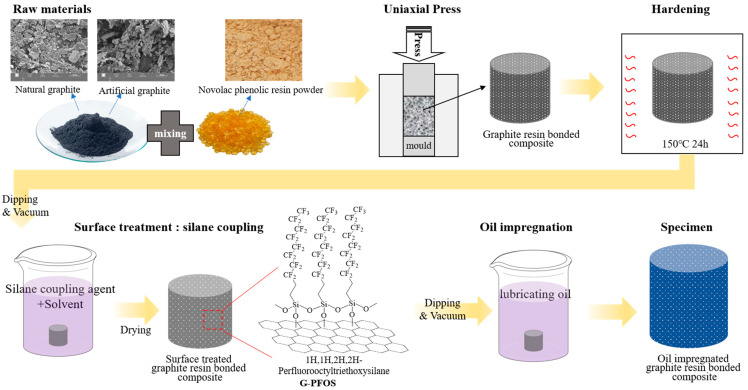
Schematic process illustration of resin-bonded solid lubricant specimen using natural graphite/artificial graphite filler and hydrophobic silane coupling.

Figure 1 illustrates the specimen fabrication process, including silane coupling treatment and lubricant oil impregnation. The specimens (graphite–resin composites) were prepared by mixing graphite powder and phenolic resin (novolac type) in a 6:4 weight ratio and molding them into cylindrical specimens with a diameter of 10 mm under a pressure of 50 MPa. The fillers were mixed in ratios of 100:0, 75:25, 50:50, 25:75, and 0:100 (natural:artificial graphite). The molded specimens were heated to 150 °C at a rate of 2 °C/min and maintained for 24 h to induce curing. For silane coupling, 1H,1H,2H,2H-Perfluorooctyltriethoxysilane (PFOS, Aldrich, St. Louis, MI, USA) was added to a 75:25 (*v*/*v*) ethanol/distilled water solution at concentrations ranging from 0.5 wt% to 3.0 wt%. The specimens were immersed in the silane coupling solution and maintained under a vacuum of 30 mtorr for 1 h to allow deep penetration. The specimens were then dried at 100 °C for 2 h to complete the silane coupling.

For oil impregnation, the silane-treated specimens were placed in a vacuum chamber and maintained under vacuum (30 mtorr) for 3 h to allow oil impregnation. The chamber was then filled with oil, and a pressure of 0.4 MPa was applied to ensure deep penetration of the oil into the specimens for 1 h. After releasing the pressure, the oil-impregnated specimens were obtained.

### 2.2. Analysis Methods

The bulk density of the specimens was calculated by dividing the weight by the volume, determined from the diameter and height measurements. Compressive strength was measured using a universal testing machine (Instron 5982, Instron, Norwood, MA, USA). The crystallinity of the graphite powder was analyzed using XRD to determine its effect on friction behavior. The XRD (MiniFlex600, d, Tokyo, Japan) conditions were as follows: *λ* = 1.5406, and the crystallinity was calculated using Equation (1): [17].

(1) $$d_{002}=\frac{\lambda }{2\sin\theta },\;\lambda =1.5406,\;\mathrm{Crystallinity}=100*\frac{3.440-d_{002}}{0.086}$$

The surface of the specimens was analyzed using Fourier transform infrared spectroscopy (FTIR, Spotlight400, PerkinElmer, Wellseley, MA, USA) in the range of 4000–400 cm^−1^ with a resolution of 4 cm^−1^. The water contact angle (WCA) was measured by placing a 15 µL water droplet on the surface at room temperature using a Phoenix-300 (Surface Electro Optics, Suwon-si, Gyeonggi-do, Republic of Korea) device. The oil impregnation rate was measured by immersing the specimens in an oil bath under 0.8 atm pressure for 2 h. The impregnation rate was calculated by dividing the weight of the impregnated oil by the initial weight of the graphite.

**Figure 2:**
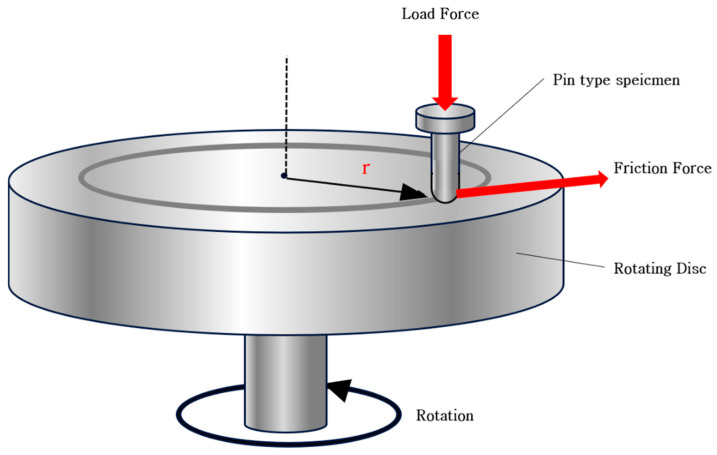
Schematic illustration of pin-on-disk friction testing for the specimen.

To investigate the frictional behavior of the fabricated specimen material, a pin-on-disk type friction test was performed. As shown in Figure 2, which illustrates the schematic diagram of the friction and wear testing apparatus used in this experiment, the setup consisted of a specimen pin and a counter disc in a pin-on-disc configuration, with a vertical load applied to the specimen. The counter material was an S45C steel disc with a diameter of 300 mm and a hardness of 85 HRC, and the tip of the specimen was polished to a rounded geometry. The friction coefficient was measured under dry contact conditions by placing the specimen on a steel disc rotating at 200 rpm and applying a load of 1 kg (9.8 N). The friction coefficient (μ) was calculated using the following Equation (2): where *F* is the frictional force (N) and *P* is the applied normal load (N) [8,18].

(2) $$\mu =\frac{F}{P}$$

**Figure 3:**
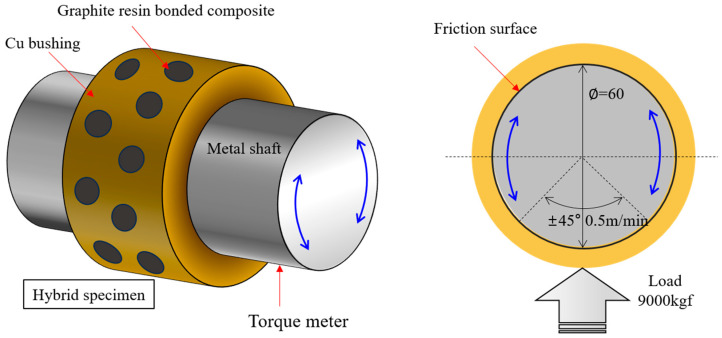
Schematic illustration of the machine used for friction testing of the specimen.

To evaluate the wear behavior of the specimen under high-load conditions, a face-on-face friction test was conducted. As illustrated in Figure 3, which depicts the schematic of the friction testing apparatus used in this experiment, the setup comprised a hybrid copper–graphite–resin composite bushing specimen and a metal shaft. The cylindrical copper bushing (inner diameter: 60 mm, outer diameter: 70 mm) was embedded with 20 pieces of graphite–resin composite (10 mm in diameter). The surface roughness of the metal shaft was maintained below 1.6 Rz. A vertical load of 88,260 N was applied to the specimen, resulting in– a surface pressure of 50 MPa. The metal shaft underwent reciprocating motion at a speed of 0.5 m/min over a ±45° range under dry contact conditions, and the torque applied to the shaft was measured. The friction coefficient (μ) was calculated using the following Equation (2) [19,20]:

(3) $$\mu =\frac{T}{p*D}\;$$

where *T* is the measured torque (N·m), *p* is the normal load (N).

The inner diameter of the hybrid copper–graphite–resin composite bushing specimens was measured after testing. The wear volume was calculated from the difference in outer and inner diameters before and after testing, and the obtained wear volume was divided by the applied normal load and the number of reciprocating cycles to determine the wear rate of the bushing specimens, as expressed in Equation (3) [21]:

(4) $$\mathrm{Wear}\;\mathrm{rate}\;=\;\frac{{\left[L\times \pi {\left(\frac{D_{o}}{2}\right)}^{2}-L\times \pi {\left(\frac{D_{i}}{2}\right)}^{2}\right]}_{before}-{\left[L\times \pi {\left(\frac{D_{o}}{2}\right)}^{2}-L\times \pi {\left(\frac{D_{i}}{2}\right)}^{2}\right]}_{after}}{P\times n}$$

where *D*_*o*_ is the outer diameter of the specimen, *D*_*i*_ is the inner diameter, *L* is the specimen width (mm), *P* is the applied normal load (N), and *n* is the number of reciprocating cycles.

The morphology of the graphite particles was investigated using a scanning electron microscope (SEM, CLARA, TESCAN, Brno, Czech Republic). After the friction test, microstructural changes on the friction surface of the specimen were examined using an optical microscope (BX53M, OLYMPUS, Tokyo, Japan), and crystallinity changes on the friction surface were analyzed via Raman spectroscopy (HR Evolution, HORIBA, Kyoto, Japan).

## 3. Results and Discussion

### 3.1. Graphite Crystallinity and Morphology: Structural Determinants of Mechanical and Tribological Performance

**Figure 4:**
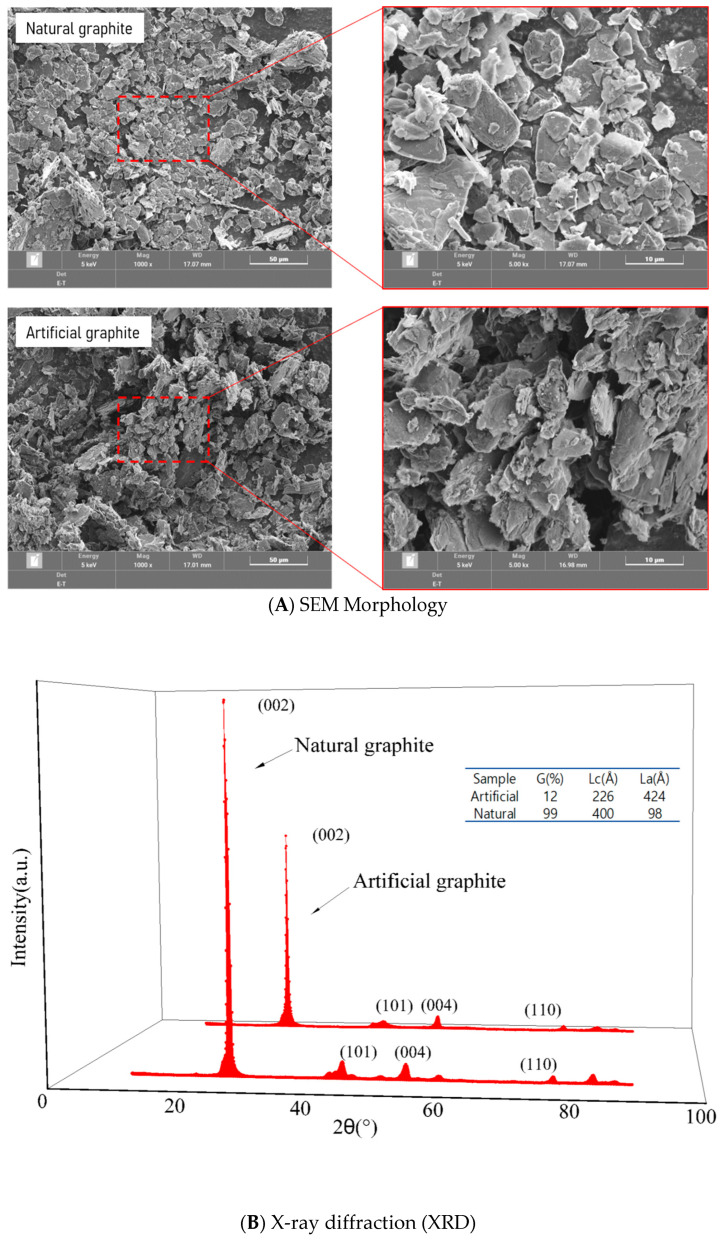
(**A**) SEM Morphology of Natural and Artificial Graphite Powders and (**B**) XRD Analysis of Graphitization Degree.

The morphology and crystal structure of graphite fillers in self-lubricating composites were analyzed using scanning electron microscopy (SEM) and X-ray diffraction (XRD). Figure 4A displays SEM images of natural graphite (NG) and artificial graphite (AG) powders at magnifications of 1000× and 5000×. NG exhibited a flaky, smooth-edged morphology, whereas AG showed irregular, fragmented particles with a porous structure. During friction, the layered structure of NG allows graphene sheets to slide and exfoliate, imparting self-lubricating properties. The morphology of graphite particles significantly influences the exfoliation behavior, thereby affecting the friction characteristics. Additionally, uniaxial compression molding during fabrication impacts the alignment of graphite particles, further modulating tribological performance.

Figure 4B presents XRD patterns of NG and AG. NG displayed a prominent (002) diffraction peak at 26–27° (2θ), indicating high graphitization (99%) with a crystal stacking height (Lc) of 400 Å and lateral crystallite size (La) of 98 Å. In contrast, AG exhibited a broader (002) peak, corresponding to lower graphitization (12%, Lc = 226Å, La = 424Å) [1,6]. The larger La in AG suggests disordered in-plane growth of graphene nano layers, characteristic of amorphous carbon structures.

**Figure 5:**
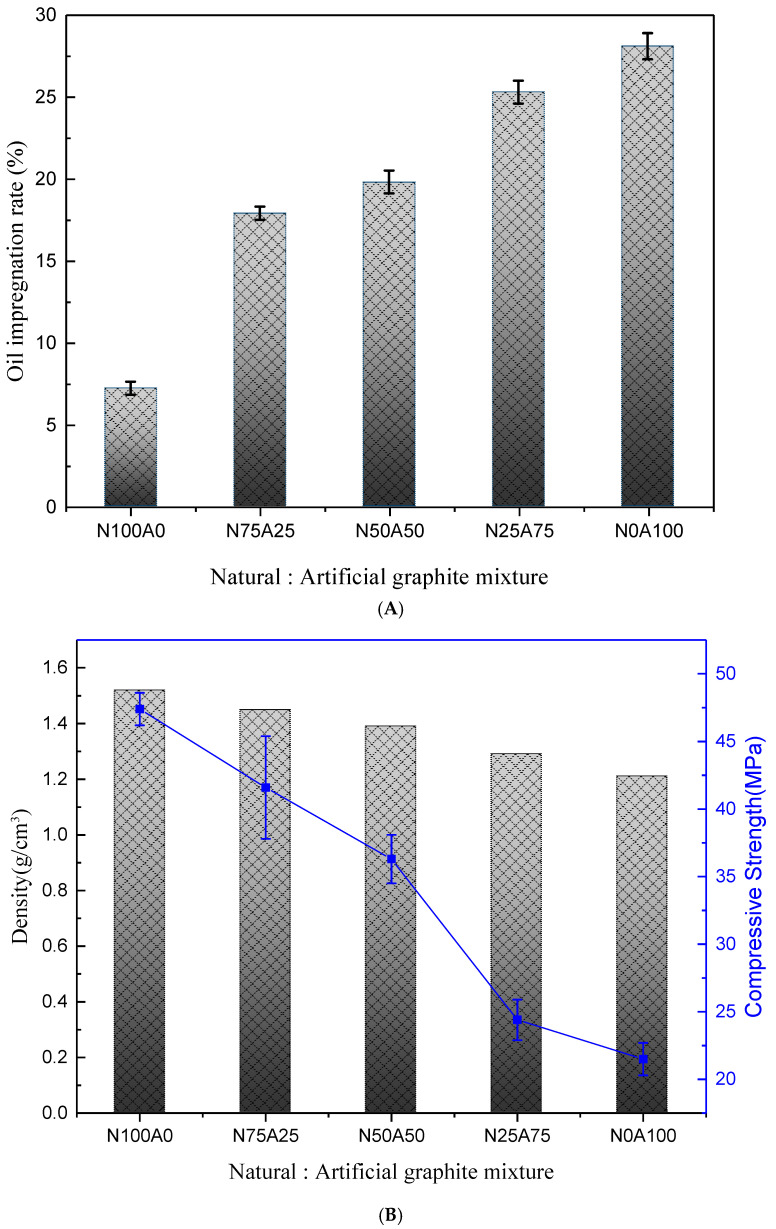
(**A**) Oil Retention Depending on the Mixing Ratio of Different Graphite Powders and (**B**) Changes in Density, Compressive Strength.

The density, compressive strength, and oil impregnation rate of graphite–resin composites were evaluated as functions of graphite blending ratios (Figure 5). Composites with higher NG content (100NG) achieved the highest density (1.92 g/cm^3^) and compressive strength (18.4 MPa) due to the anisotropic alignment of NG flakes during uniaxial compression, enabling dense stacking (Figure 5A). Conversely, AG-rich composites (100AG) exhibited lower density (1.80 g/cm^3^) and compressive strength (12.7 MPa) but superior oil impregnation (23 wt%) owing to their porous, isotropic morphology (Figure 5B) [22,23].

To evaluate the frictional behavior of the graphite–resin composites, a pin-on-disc test was conducted, and the friction coefficient was measured. The microstructure and crystallinity of the friction surfaces were further examined using scanning electron microscopy (SEM) and Raman spectroscopy [24].

**Figure 6:**
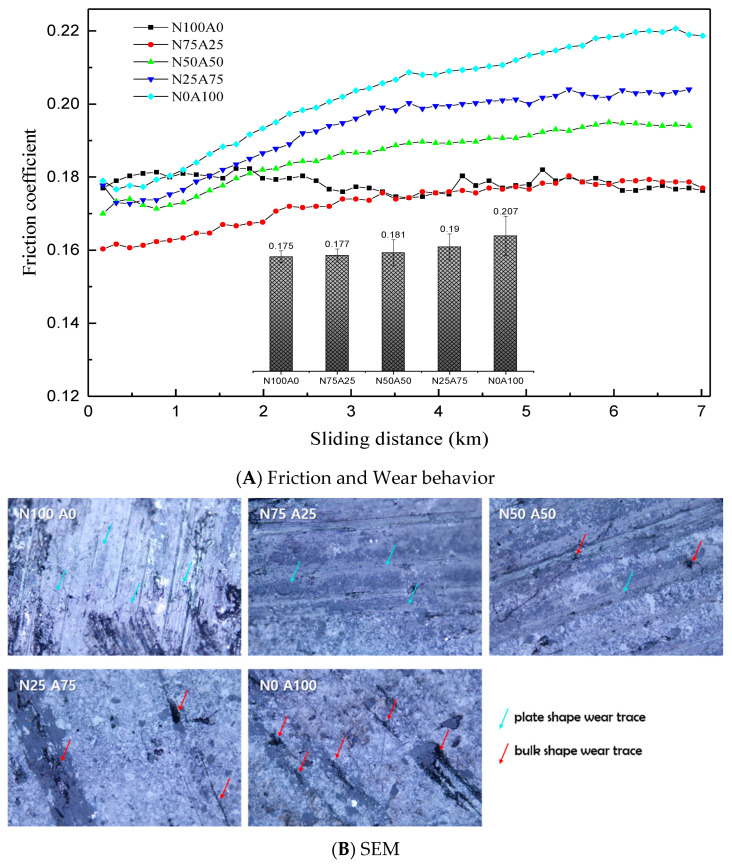

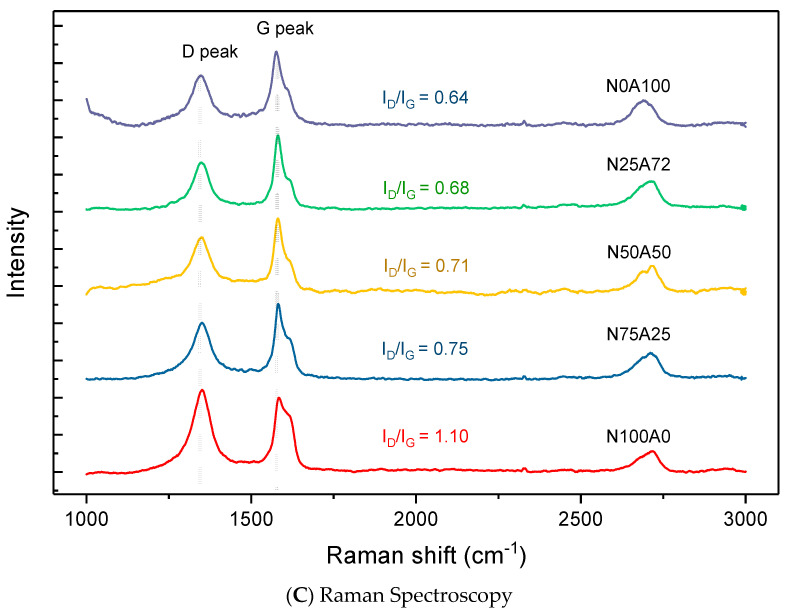
Friction and Wear Behavior (Friction Coefficient, Wear Surface Morphology, and Raman Spectroscopy) of Composites with Varying Ratios of Natural and Artificial Graphite Powders.

Figure 6A illustrates the variation in friction coefficient with sliding distance (km) for composites with different natural graphite (NG) to artificial graphite (AG) ratios. Specimens with hemispherical tips initially exhibited low friction coefficients, which gradually increased as the contact area expanded during sliding. Composites with higher NG content started with a relatively low friction coefficient, which stabilized into a steady-state value after an initial run-in period. In contrast, composites with higher AG content showed a significant increase in friction coefficient during the run-in phase, reaching the highest value in the steady state.

The stable friction behavior of NG-rich composites is attributed to the continuous exfoliation of graphene nano layers from NG particles during sliding, facilitated by their highly ordered layered structure and high compressive strength [5,8]. In contrast, AG-rich composites, with lower crystallinity and compressive strength, experienced particle detachment due to weak interfacial bonding, leading to abrasive wear and unstable friction.

Figure 6B presents SEM images of the wear tracks. NG-rich composites exhibited uniform plate-like wear traces, indicative of smooth graphene exfoliation. Conversely, AG-rich composites showed bulk wear traces with pits and cracks caused by the fragmentation and dislodgment of irregular AG particles [5]. This difference arises from NG’s flaky morphology, which promotes planar wear, while AG’s fragmented, three-dimensional particles hinder effective exfoliation, resulting in mechanical interlocking and particle pull-out.

Figure 6C displays Raman spectroscopy results, highlighting changes in crystallinity at the friction surface. NG-rich composites exhibited higher ID/IG ratios, reflecting greater graphene exfoliation and structural disorder due to shear-induced layer separation [5]. AG-rich composites showed lower ID/IG ratios, consistent with minimal exfoliation and the accumulation of disordered carbon debris from particle fragmentation.

To evaluate the frictional behavior of oil-impregnated specimens, the friction coefficient was measured using a pin-on-disc test, and the wear surface microstructure was examined via scanning electron microscopy (SEM).

**Figure 7:**
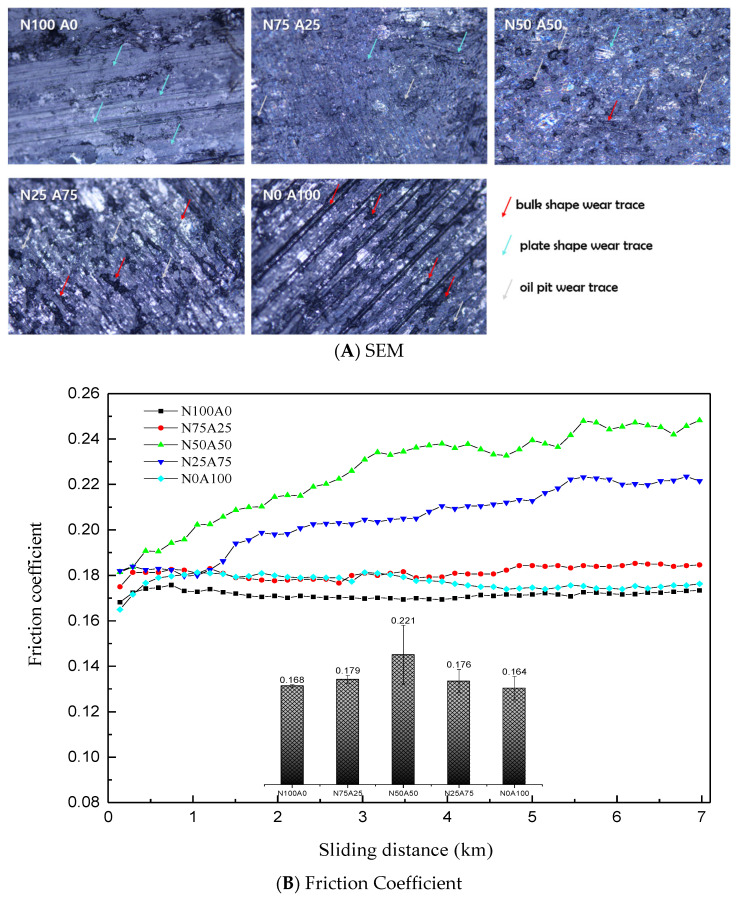
Friction Coefficient and Wear Surface Morphology of Composites with Varied Mixing Ratios of Natural and Artificial Graphite Powders.

Figure 7A illustrates the variation in friction coefficient with sliding distance (km) for composites with different ratios of natural graphite (NG) to artificial graphite (AG) after oil impregnation. Initially, specimens with the highest NG or AG content exhibited the lowest friction coefficients. These values remained low even during the steady-state phase. However, the hybrid composite (N50A50, 50% NG:50% AG) showed a continuous increase in friction coefficient until stabilization, reaching the highest value. Oil impregnation also led to the formation of oil pit wear traces on the wear surfaces.

The stable friction behavior of NG-rich composites is attributed to the smooth exfoliation of graphene nano layers from NG particles during sliding, combined with their high compressive strength, which maintains surface integrity. Additionally, the alignment of NG particles along the friction direction during uniaxial compression molding enhances stability. In contrast, AG-rich composites, despite their low friction coefficients, exhibited mechanical degradation due to low density and compressive strength [9,19]. Their porous structure facilitated higher oil retention but caused softening of the wear surface, leading to particle detachment and the formation of bulk wear traces and oil pit traces (Figure 7B).

Figure 7B presents SEM images of wear surfaces after friction testing. NG-rich composites displayed uniform plate-like wear traces, indicating controlled wear and effective lubrication from the impregnated oil. Conversely, AG-rich composites showed pronounced bulk wear traces with deep pits and cracks, resulting from particle dislodgment and abrasive interactions due to their low mechanical strength.

**Figure 8:**
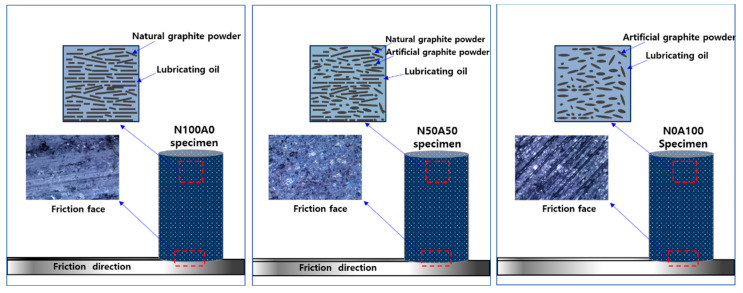
Schematic Illustration of Friction and Wear Behavior in Graphite–Resin Solid Lubricants (Pin-on-Disk Configuration).

The schematic in Figure 8 illustrates the distinct friction and wear mechanisms of graphite–resin composites based on the ratio of natural graphite (NG) to artificial graphite (AG). Composites dominated by natural graphite exhibit a dense, horizontally aligned microstructure due to the uniaxial compression molding process. This alignment allows graphene nano layers within NG to slide and exfoliate smoothly along the friction direction, forming a uniform lubrication layer. The synergy between the exfoliated graphene and impregnated oil results in stable friction behavior, characterized by low friction coefficients and minimal surface damage, as evidenced by the presence of plate-shaped wear traces.

In contrast, hybrid composites with equal parts NG and AG (50:50) display a disrupted microstructure. While NG flakes partially align during molding, the irregular, three-dimensional morphology of AG particles introduces structural heterogeneity. This loosely packed arrangement enhances oil retention but compromises mechanical stability. During sliding, two competing mechanisms dominate, graphene exfoliation from NG contributes to lubrication, while AG particle detachment generates abrasive interactions, leading to bulk-shaped wear traces (pits and embedded particles) [8,10]. These opposing effects cause fluctuations in the friction coefficient, reflecting unstable tribological performance.

Composites rich in artificial graphite exhibit a porous, disordered microstructure due to AG’s fragmented morphology. While this porosity enables high oil impregnation, the low compressive strength of AG promotes rapid particle dislodgment during friction. Detached AG particles interact with the oil, forming discontinuous lubrication zones and prominent bulk-shaped wear traces. Despite an initially low friction coefficient, the weak interfacial bonding and abrasive wear mechanisms result in erratic friction behavior over time.

This schematic underscores the critical interplay between graphite morphology, oil retention, and mechanical integrity. Natural graphite ensures stability through aligned graphene nano layers, while artificial graphite enhances lubricant storage at the cost of structural robustness. Optimizing the NG/AG ratio is essential for balancing lubrication efficiency and wear resistance in solid lubricant design.

### 3.2. Hydrophobic Silane Coupling: Optimizing Oil Retention and Lubrication Efficiency

To enhance the oil impregnation capacity of the fabricated graphite–resin composite specimens, hydrophobic silane coupling treatment was applied. Water contact angle measurements were conducted to evaluate the hydrophobicity of the surface induced by silane coupling treatment. The functional groups and wettability of the treated specimens were analyzed using FT-IR spectroscopy and water contact angle measurements.

**Figure 9:**
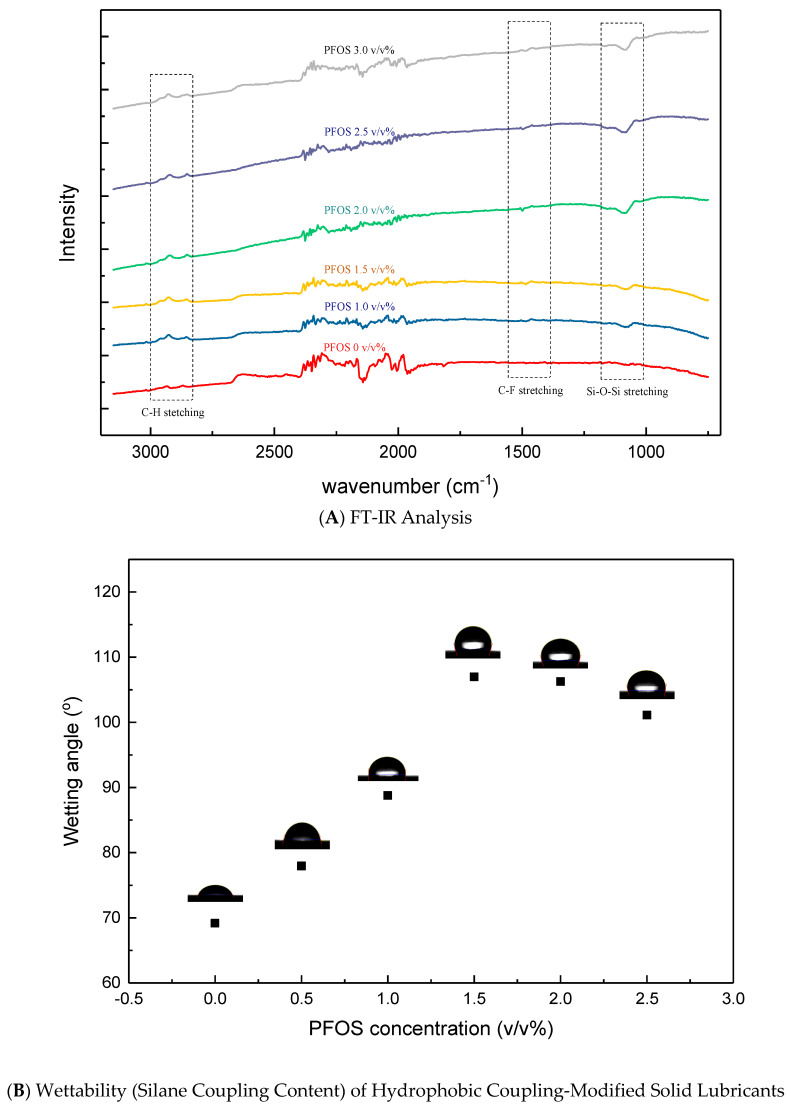
FT-IR Analysis and Wettability (Silane Coupling Content) of Hydrophobic Coupling-Modified Solid Lubricants.

Figure 9A presents the FT-IR spectra of specimens treated with varying concentrations (*v*/*v*%) of PFOS (1H,1H,2H,2H-perfluorooctyltriethoxysilane). For silane-treated graphite fillers, consistent C-H stretching peaks were observed in the 2800–3000 cm^−1^ wavenumber range. These peaks correspond to asymmetric stretching vibrations (2950–2850 cm^−1^) and symmetric stretching vibrations (3000–2960 cm^−1^) of alkyl groups. Additionally, distinct Si-O-Si stretching peaks (1100–1000 cm^−1^) and C-F stretching peaks (1200–1400 cm^−1^) were identified, confirming the successful grafting of fluorinated hydrophobic silane coupling agents onto the graphite surface [13,15,25,26].

The intensity of the Si-O-Si and C-F peaks increased with PFOS concentration up to 1.5 *v*/*v*%, beyond which no further enhancement was observed. This saturation occurs because PFOS, with its high surface energy (50–60 mN/m), readily forms a self-limiting monolayer on the graphite surface. Once a complete monolayer is achieved, additional PFOS does not contribute to chemical bonding, resulting in constant peak intensities.

Figure 9B illustrates the change in water contact angle (WCA) after PFOS treatment. The WCA increased from 70° (untreated) to 107° (PFOS-treated), confirming the transition of the graphite surface to a highly hydrophobic state [14]. As shown in Figure 6A, the WCA plateaued at PFOS concentrations above 1.5 *v*/*v*%, consistent with the formation of a saturated monolayer. This indicates that further increases in PFOS concentration do not improve wettability, as the surface modification is limited to a single molecular layer [14].

**Figure 10:**
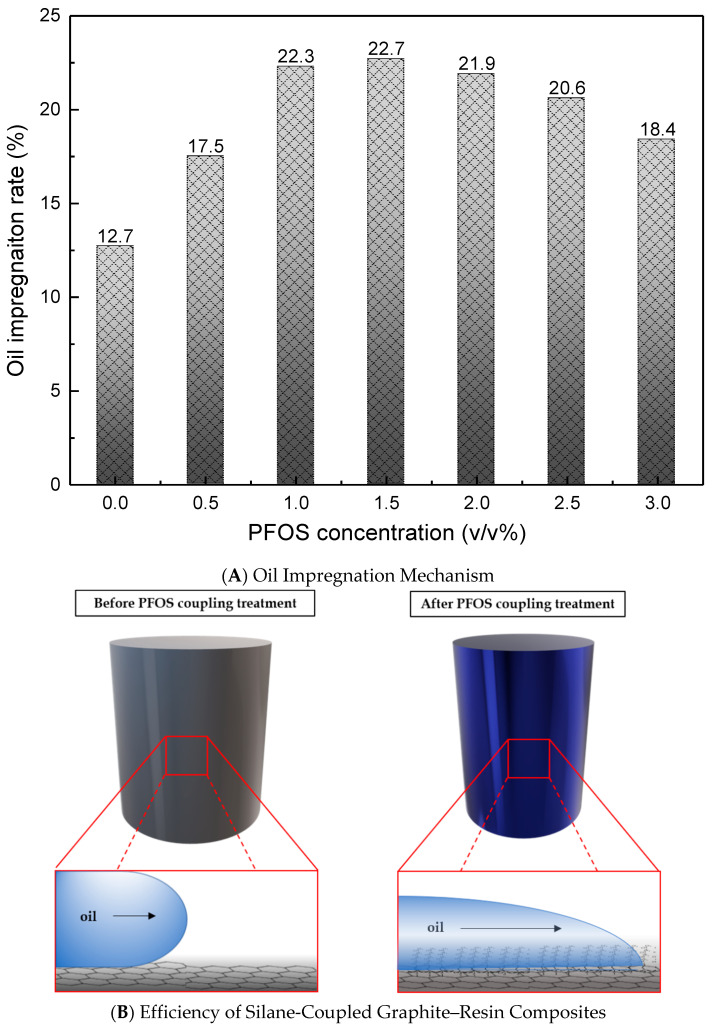
An Oil Impregnation Mechanism and Efficiency of Silane-Coupled Graphite–Resin Composites.

The oil impregnation capacity of graphite–resin composites was evaluated after hydrophobic modification with PFOS (1H,1H,2H,2H-perfluorooctyltriethoxysilane). Figure 10A quantifies the oil retention of composites as a function of PFOS concentration. Untreated composites exhibited an oil impregnation rate of 17 wt%, which increased to a maximum of 23 wt% after PFOS treatment. However, beyond the optimal PFOS concentration of 1.5 *v*/*v*%, oil retention plateaued and slightly decreased. This trend arises from the formation of a uniform hydrophobic monolayer on the graphite surface at 1.5 *v*/*v*%, beyond which excess PFOS molecules aggregate into unstable multi-layers, obstructing oil penetration.

Figure 10B schematically illustrates the oil impregnation mechanism in PFOS-modified composites. The fluorinated silane (PFOS) undergoes a condensation reaction with hydroxyl (-OH) groups on the graphite surface, releasing water (H_2_O) and forming stable C-F bonds [15,16]. This chemical grafting creates a hydrophobic interface, enhancing compatibility and wettability with nonpolar lubricating oils. Consequently, the modified surface facilitates deeper oil penetration into the composite, optimizing lubrication efficiency.

### 3.3. High-Load Tribological Behavior: Balancing Friction Stability and Structural Integrity

**Figure 11:**
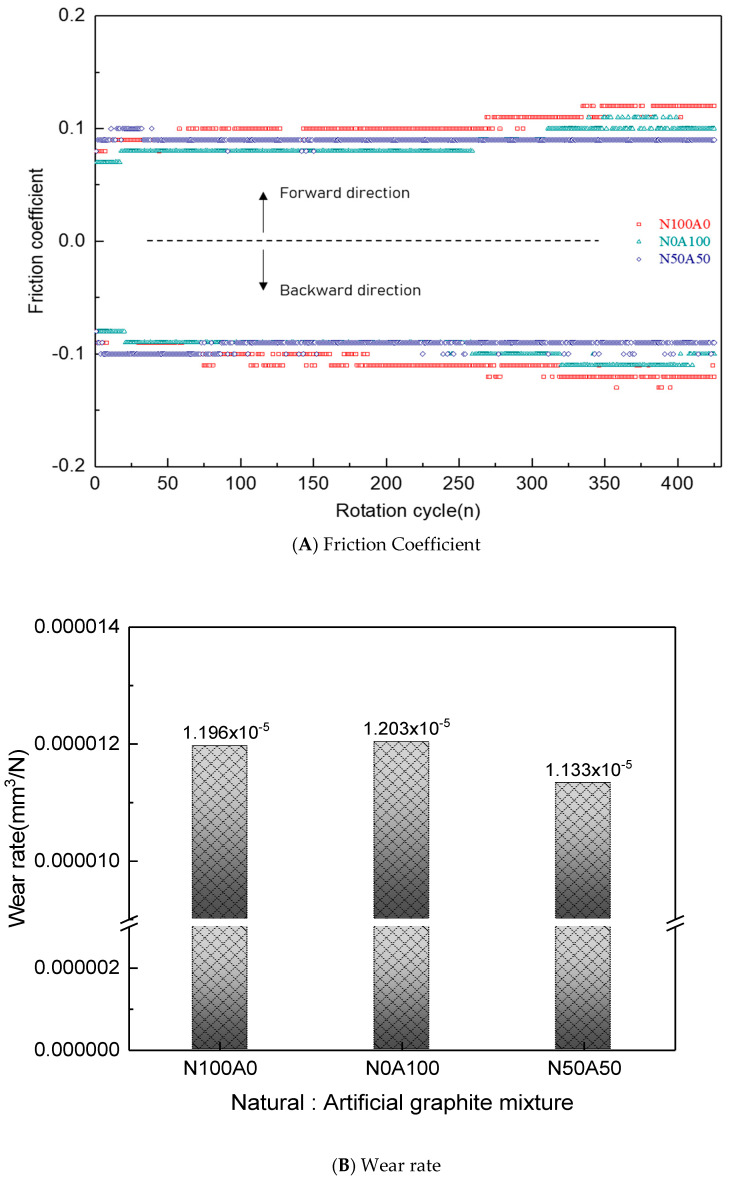
Friction Coefficient and Wear rate of Graphite–Resin Bonded Composite Hybrid Specimen with Cu Bushing as a Function of Mixing Ratio.

Figure 11A shows the friction coefficient values of hybrid specimens (graphite–resin bonded composite with Cu bushing) during reciprocating wear motion, depending on the mixing ratio of natural and artificial graphite. The N100A0 specimen (100% natural graphite) initially exhibited a low friction coefficient, but the value steadily increased as the number of cycles rose. This specimen, with high compressive strength and low oil absorption, showed low initial friction due to uniform exfoliation of graphene from natural graphite particles. However, under high-load conditions, insufficient oil absorption led to direct friction between the specimen and the counter shaft, causing instability and a subsequent rise in the friction coefficient [10]. and The N0A100 specimen (100% artificial graphite) displayed the lowest initial friction coefficient, but this value also increased with cycling. Its low compressive strength and high oil absorption allowed the formation of an oil-rich friction layer initially. However, as friction progressed, artificial graphite particles fractured and detached due to weak compressive strength, forming an unstable friction layer and increasing the friction coefficient [19,20,27].

Figure 11B presents the wear rate of the hybrid copper–graphite–resin composite bushing specimen. The wear volume results indicate that the N100A0 and N0A100 specimens exhibited relatively large wear, consistent with their unstable and continuously increasing friction coefficients. For the N100A0 specimen, the relatively low compressive strength facilitated the sliding and severe exfoliation of the layered graphite structure under frictional wear. In contrast, the N0A100 specimen, with its higher compressive strength, showed lower wear volume than N100A0; however, as shown in Figure 6, the intrinsically higher friction coefficient of artificial graphite led to particle detachment during sliding. Consequently, the specimen containing natural and artificial graphite in a 50:50 ratio demonstrated the most stable tribological performance, characterized by a low and steady friction coefficient and reduced wear volume, owing to the combined effects of sliding behavior and higher compressive strength.

In contrast, the N50A50 specimen (50% natural and 50% artificial graphite) maintained a low and stable friction coefficient. Its balanced compressive strength ensured steady graphene exfoliation from graphite particles, while moderate oil absorption promoted the formation of a stable, hybrid friction layer composed of oil and exfoliated graphene. This combination effectively sustained low and consistent friction behavior throughout the test [10,15].

**Figure 12:**
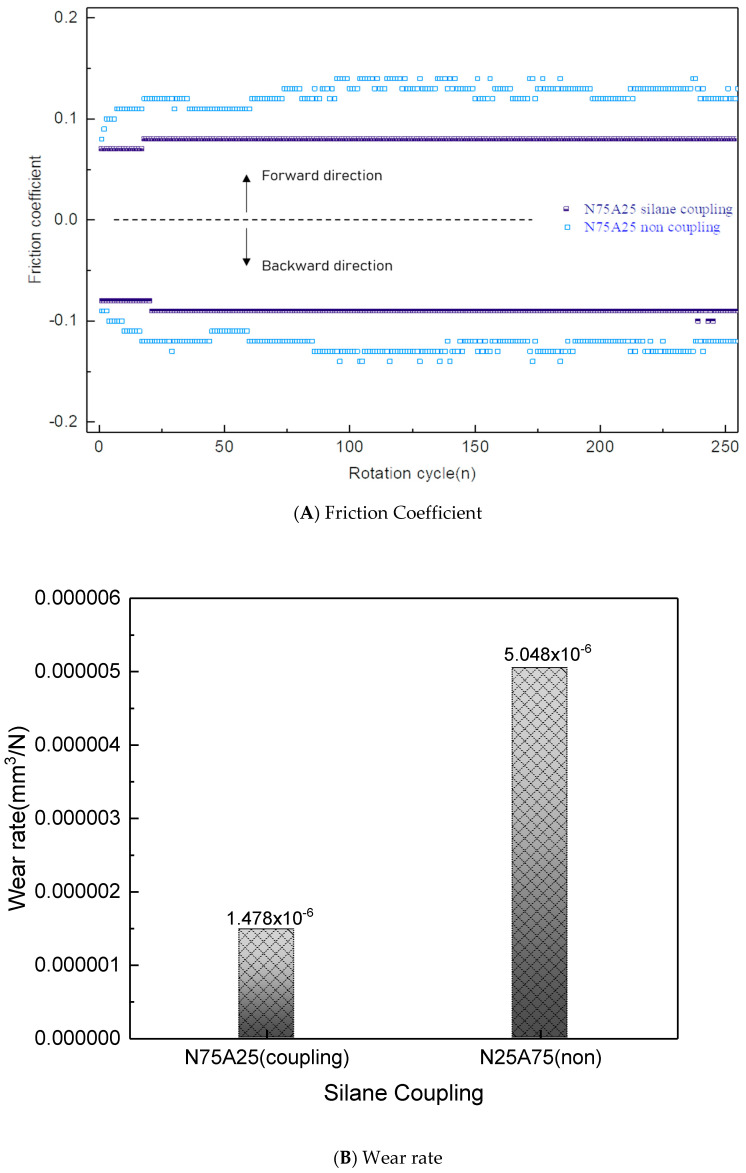
Friction Coefficient and Wear rate of Graphite–Resin Bonded Composite Solid Lubricant via Hydrophobic Silane Coupling.

Figure 12A shows the friction coefficient values of a hybrid specimen (composed of 75% natural graphite and 25% artificial graphite) combined with a Cu bushing during reciprocating wear motion, depending on silane coupling treatment. The friction coefficient was measured before and after hydrophobic PFOS treatment. The untreated specimen exhibited an increasing and then stabilizing friction coefficient, but with unstable fluctuations [14,16]. In contrast, the PFOS-treated specimen demonstrated a low friction coefficient from the initial stage, maintaining stable and low values throughout the friction process. As shown in Figure 7A, the hydrophobic PFOS treatment enhanced oil absorption by over 30 wt%, which contributed to this behavior. The increased oil absorption, along with graphene exfoliated from natural graphite particles during friction, formed a stable lubrication layer, resulting in low and consistent friction. Additionally, the high compressive strength of the hybrid specimen suppressed cracking or particle detachment even under high-load friction conditions [15,19].

Figure 12B presents the wear rate of the hybrid copper–graphite–resin composite bushing specimen. The comparison between pre- and post-impregnation specimens also revealed a significant difference in wear volume. The specimens without silane coupling treatment showed relatively high wear, whereas those with silane coupling treatment exhibited markedly lower wear. This behavior can be attributed to the lubrication mechanism: as observed in the friction coefficient evolution (Figure 12A), the initial frictional wear generated stable wear tracks, enabling the relatively abundant impregnated lubricant to be supplied in a stable manner, thereby reducing friction and suppressing surface exfoliation. In contrast, specimens without silane coupling treatment contained a lower amount of impregnated lubricant; thus, after the formation of wear tracks, direct abrasion of graphite particles occurred at the worn surfaces, leading to higher wear volume.

## 4. Conclusions

This study successfully elucidated the effects of graphite filler crystallinity, nano morphology, and hydrophobic coupling treatment on improving the friction and wear behavior of graphite–resin bonded composites. By controlling the blending ratio of highly crystalline, flaky natural graphite and low-crystalline, three-dimensional artificial graphite, the correlation between compressive strength and oil absorption was identified, enabling the fabrication of an optimized graphite–resin composite with superior friction performance. Additionally, hydrophobic coupling treatment of the composite enhanced compatibility with hydrophobic lubricants, significantly improving oil absorption within the specimen. Flaky natural graphite, with its densely packed structure, exhibited high compressive strength but low oil absorption, resulting in a relatively high initial friction coefficient. However, as friction progressed, it maintained a consistently low and stable friction coefficient. Three-dimensional artificial graphite, with its loosely structured morphology, showed low compressive strength but high oil absorption, leading to a low initial friction coefficient. However, unstable friction behavior and a gradual rise in friction coefficient were observed as friction progressed due to particle detachment and structural degradation.

Hydrophobic coupling treatment promoted oil penetration into the composite by ensuring compatibility with hydrophobic lubricants, achieving high oil absorption. This directly contributed to low, stable friction behavior, attributed to the formation of a durable lubricating layer during friction. This layer effectively reduced surface damage and preserved the microstructural integrity of graphite particles under wear. Moreover, dispersed copper bushing particles on the graphite surface facilitate the formation of a lubricating layer, resulting in a reduced friction coefficient [28].

Under high-load conditions (3530 N, 3 MPa surface pressure), Natural graphite maintained stable friction behavior due to its high compressive strength and dense microstructure, but exhibited elevated friction coefficients. Artificial graphite, with its weak compressive strength and porous microstructure, initially showed low friction due to high oil absorption. However, its fragile structure led to severe damage as friction progressed, causing a sharp increase in friction coefficient and wear rate. Detached artificial graphite particles further induced unstable wear behavior through direct contact with the metal shaft.

These findings highlight the critical balance between graphite nano morphology, oil absorption, and mechanical strength in designing high-performance, wear-resistant graphite–resin composites for demanding tribological applications.

As part of future work, the high-load wear performance and porous microstructure will be systematically investigated.

## Figures and Tables

**Table 1:** General properties of lubricant filler, lubricant oil, and binder.

Lubricant Filler				
**Graphite Powder**	**Density (g/cm^3^)**	**Particle Size (μm)**	**Direction**	**Company**
Natural Graphite	1.92	110.5	anisotropic	CHINA Q
Artificial Graphite	1.80	30.8	isotropic	USA I
**Lubricant Oil**				
**Impregnation Liquid**	**Density (g/cm^3^ at 15 °C)**	**Viscosity (mm^2^/s at 40 °C)**	**Flash Point (°C)**	**Company**
RUBIA 6800 15W-40	0.876	102.8	232	S-OIL
**Binder**				
**Novolac Phenolic Resin**	**Molecular Weight (Mw)**	**Softening Temp. (°C)**	**Hardening Time (sec)**	**Company**
KC3002	1300~1700	113	85–120	KOREA K

## Data Availability

The original contributions presented in this study are included in the article material. Further inquiries can be directed to the corresponding author.
